# Multi-Element Composition of Diatom *Chaetoceros* spp. from Natural Phytoplankton Assemblages of the Russian Arctic Seas

**DOI:** 10.3390/biology10101009

**Published:** 2021-10-08

**Authors:** Nikolay V. Lobus, Maxim S. Kulikovskiy, Yevhen I. Maltsev

**Affiliations:** Laboratory of Molecular Systematics of Aquatic Plants, Timiryazev Institute of Plant Physiology, Russian Academy of Sciences (IPP RAS), Botanicheskaya St. 35, 127276 Moscow, Russia; max-kulikovsky@yandex.ru (M.S.K.); ye.maltsev@gmail.com (Y.I.M.)

**Keywords:** Arctic, chemical composition of phytoplankton, microalgae, bioaccumulation, biogenic silica, mineral nutrients, trace elements, rare earth elements

## Abstract

**Simple Summary:**

Despite the long history of studying the elemental composition of phytoplankton and its individual ecological and systematic groups or specific algae species, the global dataset is far from completed. Our original research aims to study the elemental composition of a certain taxonomic group of marine diatoms, whose representatives make a significant contribution to primary production in the Arctic Ocean. The data on the chemical composition of diatom microalgae are discussed concerning their role in the global biogeochemical circulation of elements in the ocean. In particular, the obtained data make a prominent input to the study of the multi-element composition of marine diatom species, namely *Chaetoceros* spp., inhabiting the shelf seas of the Arctic Ocean. These data may be used as a basis for the cultivation of marine diatom strains for obtaining commercially promising producers of biogenic silica or valuable biological products that can be used as raw materials in the production of feed and nutrition for agriculture and aquaculture.

**Abstract:**

Data on the elemental composition of the diatom *Chaetoceros* spp. from natural phytoplankton communities of Arctic marine ecosystems are presented for the first time. Samples were collected during the 69th cruise (22 August–26 September 2017) of the R/V Akademik Mstislav Keldysh in the Kara, Laptev, and East Siberian Seas. The multi-element composition of the diatom microalgae was studied by ICP-AES and ICP-MS methods. The contents of major (Na, Mg, Al, Si, P, S, K and Ca), trace (Li, Be, B, Ti, V, Cr, Mn, Co, Ni, Cu, Zn, Ga, As, Se, Rb, Sr, Mo, Ag, Cd, Sn, Sb, Cs, Ba, Hg, Tl, Pb, Bi, Th and U) and rare earth (Sc, Y, La, Ce, Pr, Nd, Sm, Eu, Gd, Tb, Dy, Ho, Er, Tm, Yb, and Lu) elements varied greatly, which was probably associated with the peculiarities of the functional state and mineral nutrition of phytoplankton in the autumn period. Biogenic silicon was the dominant component of the chemical composition of *Chaetoceros* spp., averaging 19.10 ± 0.58% of dry weight (DW). Other significant macronutrients were alkaline (Na and K) and alkaline earth (Ca and Mg) metals as well as biogenic (S and P) and essential (Al and Fe) elements. Their total contents varied from 1.26 to 2.72% DW, averaging 2.07 ± 0.43% DW. The Al:Si ratio for natural assemblages of *Chaetoceros* spp. of the shelf seas of the Arctic Ocean was 5.8 × 10^−3^. The total concentrations of trace and rare earth elements on average were 654.42 ± 120.07 and 4.14 ± 1.37 μg g^−1^ DW, respectively. We summarize the scarce data on the average chemical composition of marine and oceanic phytoplankton and discuss the limitations and approaches of such studies. We conclude on the lack of data and the need for further targeted studies on this issue.

## 1. Introduction

Phytoplankton is an integral component of marine ecosystems and plays a key role in the biogeochemical cycles of major and trace elements in the ocean [1,2,3,4]. Interacting directly with the dissolved forms of chemical elements through adsorption, desorption, and biological absorption, phytoplankton effectively extract these substances from the environment, involving them in biogenic cycles [5,6]. The assimilation of elements by phytoplankton radically changes their geochemical pathways in the ocean, providing a multiple increase in the residence time in the water column and promoting their transfer to higher trophic levels [7,8,9]. The coordination of biological and geochemical processes is a result of the biogeochemical evolution of ecosystems over millions of years [3,10,11]. On the one hand, the dynamics of the contents of chemical elements in the euphotic zone of the ocean is closely related to large-scale biological cycles of primary producers [4,9,12]. On the other hand, some of the major and trace elements are important co-factors of biochemical and physiological processes in the algae cell [11,13]. Therefore, they control the growth rates of certain species, precondition the overall succession of the phytoplankton community, and regulate its taxonomic structure [1,3,11,14,15].

Data on the chemical composition of phytoplankton and its certain taxonomic groups are scarce [5,7,14,16]. First of all, this is due to the technical difficulties in collecting and obtaining pure samples of natural phytoplankton communities containing a minimum amount of mineral admixtures [17,18]. However, according to recent studies, analyzing the multi-element composition of phytoplankton is important both for assessing the dynamics and fluctuations of marine ecosystems in changing climatic conditions [3,9,11,19,20] and for the development of theoretical foundations of the safety of using microalgae as potential natural sources of functional food [21,22,23,24].

In marine ecosystems of high latitudes, *Chaetoceros* Ehrenberg (Bacillariophyta) is one of the most numerous and widespread genera of planktonic diatoms. Being the most important functional component of pelagic ecosystems, the species of the genus *Chaetoceros* may form powerful blooms and serve as a food directly for zooplankton in the water column and indirectly as a source of organic carbon for the benthic communities [25,26]. In addition, intensive development of the *Chaetoceros* spp. diatoms in the Arctic Ocean has a significant impact on the biogeochemical cycle of carbon and silicon, as well as on a wide range of macronutrients and trace elements [26,27,28].

The presence of long, thin outgrowths (spines), aiming to reduce the sinking rate of a cell, is a distinctive morphological feature of representatives of this genus. Cell sized vary over a wide range of 2–3 µm up to 50 µm and even more. Spines, connecting the frustules of the cell, promote the formation of colonies, which may comprise up to several hundred cells and may reach a length of several millimeters [29]. Some *Chaetoceros* species are well-established commercial aquacultures [30,31,32]. Many of them are recognized as generally good producers of useful lipids and other biologically active products with high value-added. They have enormous potential for producing nutraceuticals and biofuel [24,33,34].

The study aims to analyze the multi-element composition of natural communities of diatom phytoplankton represented by species of the genus *Chaetoceros* spp., inhabiting the shelf seas of the Russian Arctic.

## 2. Materials and Methods

### 2.1. Environmental Setting

During the sampling period, the thermohaline structure of the water masses of the Kara, Laptev, and East Siberian seas was heterogeneous and was characterized by pronounced vertical stratification [35,36,37]. It was associated with the freshwater runoff of the largest rivers of Western and Eastern Siberia, where almost ubiquitous desalination of the surface layer was observed down to less than 28 practical salinity units (PSU). The upper, warmer layer of desalinated water, characterized by temperature of +1.78–+2.55 °C and a salinity of 22–28 PSU at station no. 5625 (Laptev Sea) and +3.46–+3.72 °C and 25–28 PSU at station no. 5587/2 (Kara Sea), occupied the surface water layers of 0–9 m and 0–15 m, respectively [35]. It was separated from the lower layers by a sharp halocline, under which colder (down to −1.77 °C) and saltier (30–34 PSU) water masses were located. The pattern was different for the eastern part of the East Siberian Sea, including station no. 5612 [38]. Vertical changes in water salinity, density, and temperature were less pronounced. Obviously, in this area, the seasonal cooling of the surface layer caused vertical convection that affected the entire water column [39,40].

In September 2017, the hydrochemical structure of the waters in the studied areas of the Arctic seas of Russia corresponded to that described for the autumn period [39,40,41]. An extremely low content of nitrate nitrogen was observed at all the studied sites; this was the main factor limiting seasonal primary production and phytoplankton development. As a result, the abundance and biomass of phytoplankton was low, namely 50–75 × 10^3^ cells/L and ~50 mg wet weight/m^3^, respectively [39]. Phytoplankton abundance reaching 300–400 × 10^3^ cells/L and biomass of 600–700 wet weight/m^3^ was observed in the areas belonging to the inner shelf, where a significant influence of river runoff was manifested [37,39]. It was previously reported that in the autumn on the outer shelf of the Arctic seas, the local input of nutrients into the euphotic layer might lead to the abundant development of diatom microalgae [42] and the formation of “blooming spots” characterized by increased abundance and biomass [37] against the background of seasonal vanishing of the development of the phytoplankton community.

Overall, in the shelf seas of the Arctic Ocean, the productivity and composition of phytoplankton communities are jointly governed by strong seasonality in the light regime and sea-ice cover, as well as a strong freshwater signal originating from river runoff [43,44,45].

### 2.2. Field Studies

**Figure 1:**
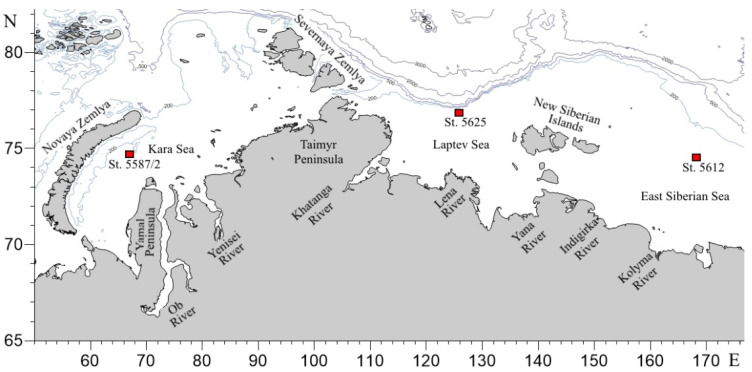
Sampling site map.

Samples were collected during the 69th cruise (22 August–26 September 2017) of the R/V Akademik Mstislav Keldysh in the Kara, Laptev, and East Siberian Seas in September 2017 (Figure 1). Sampling was performed at stations located at a maximum possible distance from the shore in areas least affected by the freshwater runoff of large rivers of Siberia and characterized by the presence of the diatom microalgae *Chaetoceros* spp. A summary of the stations is provided in Table 1 [35,38,40,41].

**Figure 2:**
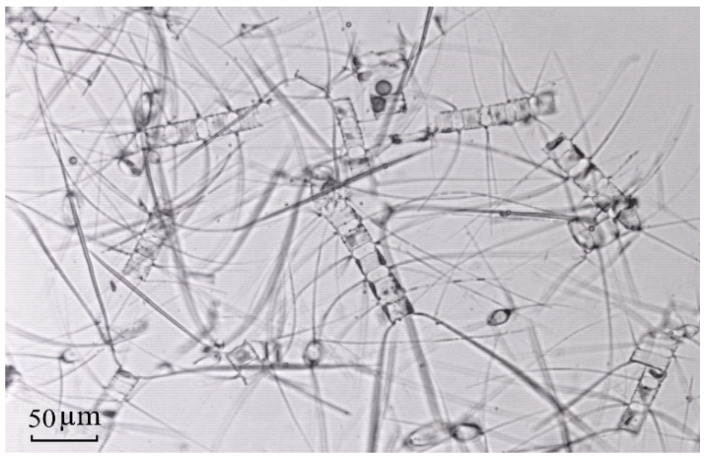
Microphotograph of the sample of diatoms of *Chaetoceros* spp. from the East Siberian Sea (station no. 5612).

Samples were taken with a standard Juday plankton net (mouth area 0.1 m^2^, mesh size 180 μm) by vertical trawling at a speed of 0.6–0.8 m sec^−1^ in the upper 45-m water layer. Three samples were taken at each station. The 180-μm mesh allowed us to collect only large phytoplankton species, mainly of the genus *Chaetoceros*, and to ensure we obtained samples devoid of a large amount of mineral suspension. In the onboard laboratory, the samples were concentrated and immediately placed in 1 L containers filled with pre-filtered (Millipore membrane nuclear filters of 47 mm diameter and 0.45 μm pore size, Merck Millipore, Burlington, MA, USA) and artificially CO_2_-saturated seawater. Subsequently, the samples were gently mixed and placed in a refrigerator (+4 °C) for ~3–5 h. Carbon dioxide dissolved in water provided instant anesthesia of all size groups of zooplankton, albeit without a toxic effect on microalga cells. Subsequent sedimentation of the sample contributed to its stratification when dead/tranquilized zooplankton organisms and the mineral suspension particles settled to the bottom, but the living phytoplankton cells concentrated in the upper layer of the water column. Then, phytoplankton samples were concentrated and examined under a Leica binocular stereomicroscope (Leica, Wetzlar, Germany) to remove large aggregates of suspended organ–mineral detritus. Such sample preparation procedure provided pure samples of *Chaetoceros* spp. (Figure 2), which were concentrated on a nylon sieve (20 μm mesh size), washed twice with distilled water, and once with Milli-Q water. Excess water was removed with filter paper, and the sample was placed in containers made of high-quality plastic and then hermetically sealed and frozen at −25 °C. In a shore laboratory, the samples were lyophilized for 48 h (condenser temperature −85 °C, vacuum 1.0 mbar). The residual moisture content did not exceed 1.5%. Samples were stored in sealed containers at −15 °C prior to analysis.

### 2.3. Analytical Methods

The analysis of chemical elements was carried out at the Analytical Certified Centre of the Institute of Microelectronics Technology Problems and High Purity Materials of the Russian Academy of Sciences (Chernogolovka, Russia). The contents of major, trace, and rare earth elements in solutions obtained after digestion of the samples were determined by atomic emission spectrometry (ICP-AES) and inductively coupled plasma mass spectrometry (ICP-MS). The ICP-AES analyses were performed using an iCAP-6500 Duo spectrometer (Thermo Fisher Scientific, Waltham, MA, USA) and an ICP-MS, X-7 quadrupole mass spectrometer (Thermo Fisher Scientific, Waltham, MA, USA). Sample digestion was carried out in a closed system using an Ankon-AT-2 autoclave (Scientific Production Company Ankon-AT, Moscow, Russia).

A 15–20 mg weighed portion was placed in a Teflon reaction chamber, and 0.05 mL of a solution of a mixture of isotopic labels containing 8 mg L^−1^ ^146^Nd, 5 mg L^−1^ ^161^Dy, and 3 mg L^−1^ ^174^Yb was added. This solution was used to control the sample digestion by the “added–found” method. Then, 2 mL of HF (hydrofluoric acid 40% GR, ISO, Merck, Kenilworth, NJ, USA) and 0.5 mL of HNO_3_ (nitric acid 65%, max 0.0000005% Hg, GR, ISO, Merck, Kenilworth, NJ, USA) were added, and the mixture was covered with a lid and left at room temperature for 6 h. Subsequently, the chambers were placed on a hotplate, heated up to 170–180 °C, and the solution was evaporated to dryness. After cooling, 2 mL of HF, 0.5 mL of HClO_4_ (perchloric acid fuming 70% Supratur, Merck, Kenilworth, NJ, USA) and 0.2 mL of HNO_3_ were added to each chamber. The reaction chambers were sealed and fixed in the titanium body of the autoclave, and stepwise heating was carried out according to the following scheme: 160 °C (60 min), 180 °C (60 min), and 200 °C (60 min). The pressure inside the reaction chamber was ~16 MPa. After cooling, 1 mL of HNO_3_ and 1 mL of HCl (hydrochloric acid fuming 37% GR, ISP, Merck, Kenilworth, NJ, USA) were added to each sample. The reaction chambers were sealed and kept at a temperature of 160 °C for 60 min [46,47].

The Hg content was determined in separately prepared samples. For this, a 15 mg portion was treated for 30 min at 96 °C with a mixture of HCl + HNO_3_ (3:1 by volume) in an open system [4].

After cooling, all resulting solutions were transferred to polyethylene Eppendorf cups (Labcon, Petaluma, CA, USA and Deltalab, Barcelona, Spain) and 0.2 mL of 10 mg L^−1^ In solution was added, which was used as an internal standard in mass-spectral measurements. Then, the sample was brought to a volume of 10 mL using Milli-Q water. The solutions obtained by carrying out the above procedures without a sample portion were used as controls. Deionized water with a resistivity of 18.2 MΩ (Milli-Q) was used. Calibration curves were plotted using multi-element and single-element standard solutions (High-Purity Standards, North Charleston, SC, USA). The analytical procedures for elemental analysis are detailed in [47]; those for an autoclave digestion system are described in [48,49].

The ICP-AES method was applied to determine major (Na, Mg, P, S, K, and Ca) and some trace elements (Li, B, Al, Ti, V, Cr, Mn, Co, Fe, Ni, Cu, Zn, Sr, and Ba). The ICP-MS method was used to determine only trace (Li, Be, B, Sc, V, Cr, Mn, Co, Ni, Cu, Zn, Ga, As, Se, Rb, Sr, Mo, Ag, Cd, Sn, Sb, Cs, Ba, Re, Au, Hg, Tl, Pb, Bi, Th, and U) and rare earth elements (Y, La, Ce, Pr, Nd, Sm, Eu, Gd, Tb, Dy, Ho, Er, Tm, Yb, and Lu). The simultaneous use of two independent analysis methods improves the quality and accuracy of the results obtained. First, the list of the analyzed elements expands significantly. Second, an additional inter-method control of the measurement accuracy is performed for each sample when certain elements (Li, B, V, Cr, Mn, Co, Ni, Cu, Zn, Sr, and Ba), whose contents in the sample are reliably determined by both methods (ICP-AES and ICP-MS), serve as internal standards to check method accuracy [47]. The measurement results are presented for major elements as percentage of dry weight (DW) and for trace and rare earth elements as μg g^−1^ DW.

To determine the concentration of biogenic silicon (BSi) in the samples, it was pre-extracted with 2 M Na_2_CO_3_ solution at +85 °C for 5 h according to the standard method [20]. The uncooled samples were immediately centrifuged for 10 min at 4500 rpm; the obtained supernatant was taken, transferred into polyethylene Eppendorf cups, cooled, brought to a volume of 20 mL with Milli-Q water, and stored in a refrigerator at +4 °C until analysis. The Si content in the solution (% Si per DW of the sample) was determined by the ICP-AES method [47] during the first day after its extraction from the sample. The relative standard deviation was calculated for three sets of triplicates of *Chaetoceros* spp., making 5.3% of the mean.

Precision and validity of the obtained elemental analysis data were evaluated using certified standard samples: Canadian Pondweed GSO 8921–2007 EK-1 (Vinogradov Institute of Geochemistry, Siberian Branch of Russian Academy of Sciences, Irkutsk, Russia), Oriental Basma Tobacco Leaves INCT-OBTL-5 (LGC, Wesel, Germany) and Polish Virginia Tobacco Leaves INCT-PVTL-6 (LGC, Wesel, Germany), which were randomly distributed in each analyzed series [50]. The discrepancy between the certified and measured contents of elements was within the confidence intervals in all cases (Table 2). The detection limit (D/L) for all elements was calculated as described elsewhere [47] (Table 2).

The data were statistically processed in the Statistica 10.0 and Microsoft Excel 2010 software package and presented as mean and standard error (m ± SE).

## 3. Results

In autumn, in the Arctic seas of Russia, the chemical composition of the *Chaetoceros* spp. diatoms was represented mostly by biogenic silicon (BSi). Its concentration varied from 18.11 to 20.12% DW, averaging 19.10 ± 0.58% DW (Table 3). Other, most significant macronutrients were alkaline (Na and K) and alkaline earth (Ca and Mg) metals, as well as biogenic (S and P) and essential (Al and Fe) elements. However, their total content was almost an order of magnitude lower than the BSi concentration and averaged 2.07 ± 0.42% DW. In total, Na, Mg, Al, Si, P, S, K, and Ca accounted for up to a quarter of the dry weight of *Chaetoceros* spp. (Table 3). A relatively high content of Al and Fe (average concentration 0.11 ± 0.04 and 0.19 ± 0.08% DW, respectively) was a distinctive feature of the multi-element composition of the diatom *Chaetoceros* spp.

When comparing the trace element composition of *Chaetoceros* spp., a high variation in the accumulation of a wide range of elements was observed. There were differences in both individual and group bioaccumulation. The total content of trace elements from Li to U varied from 423.07 μg/g DW in *Chaetoceros* spp. from the Kara Sea up to 825.83 mg/g DW in *Chaetoceros* spp. from the Laptev Seas, averaging 654.42 ± 120.07 μg/g DW for the Siberian Arctic seas (Table 3).

Similar patterns of variability of individual and group accumulation were obtained for REE. Their total concentration in *Chaetoceros* spp. varied from 1.73 μg/g DW in the Kara Sea to 6.47 μg/g DW in the Laptev Sea, averaging 4.14 ± 1.37 mg/g DW (Table 3). The accumulation of REEs in the *Chaetoceros* spp. diatoms followed general regularities of ratios of this group of elements in various components of the environment. The total concentration of light rare-earth elements (La, Ce, Pr, Nd, Sm, and Eu) was always 4.5–5.0 times higher than the total concentration of heavy rare-earth elements (Gd, Tb, Dy, Y, Ho, Er, Tm, Yb, and Lu). At the same time, the most naturally common elements (La, Ce, and Nd) accounted for ~60% of the total content of all REEs in *Chaetoceros* spp. (Table 3).

## 4. Discussion

### 4.1. Biogenic Silica

A distinctive feature of the cell structure of all diatoms is the presence of external frustules formed by BSi; their concentration varies significantly in different species. Within a single species, the BSi content may also vary greatly and depends both on abiotic environmental factors, such as water temperature, water salinity, light, and nutrient availability, and on biotic factors, such as cell size and stage of the cell cycle [51]. The obtained values (19.10 ± 0.58% DW) are some of the few data on direct measurements of BSi content in natural communities of diatoms and, in fact, the first for the Arctic region [20].

This raises certain difficulties in comparing our data with the literature, since the available information is focused mainly on studies of algal cultures [52,53] or represents theoretically calculated BSi concentrations in diatom cells from natural phytoplankton communities [54]. The data available in the literature indicate significant differences in the estimates of the BSi contents in diatom cells, which are associated with species differences, measurement methods, and attempts to extrapolate the results of laboratory studies of cultures to natural communities of microalgae. For example, the concentration of BSi in diatoms of the Great Lakes of North America, calculated on the basis of morphometric methods, varied from 18.7 to 36.5% DW, averaging 28.05% DW [54]. However, according to other data, the BSi content in freshwater species is lower and varies from 9.82 to 29.9% DW [55]. Significant interspecific and intraspecific differences are also characteristic of marine diatoms, which are reflected in seasonal fluctuations in the BSi content in natural phytoplankton communities. In different groups of microalgae in Monterey Bay (California, USA), the BSi concentration throughout the year varies from 2.34 to 12.62% DW, with an average of 5.67% DW [5].

The total BSi concentration in the cells of different diatom species may have a wide range (≤10^3^); even within a single species, it may vary in an order of magnitude [53,56]. This indicates a high intraspecific and interspecific variability of the BSi content, which depends on a large number of biotic and abiotic environmental factors [57]. For example, when seven strains of microalgae (*Amphiprora paludosa, Cyclotella cryptica, C. meneghiniana, Navicula incerta, Nitzschia laevis, Thalassiosira guillardii,* and *T. weissflogii*) were cultivated under laboratory conditions, the change in illumination intensity resulted in six-fold variation in BSi content between species and twofold variation within one species, even if the concentration and ratio of nutrients were stable and optimal [58]. A similar dependence of BSi accumulation on environmental factors was observed in different species of diatoms, namely on temperature [52,59], concentration of macronutrients [60,61,62], essential elements [63,64], and various metals [65,66].

Comparing the ecological aspects of BSi accumulation in diatoms from freshwater and marine ecosystems, a general pattern is observed. Overall, marine diatoms contain, on average, 10 times less BSi than freshwater species [67]. Salinity is one of the most important factors of BSi accumulation in marine diatoms, as proved experimentally with *T. weissflogii* and *N. salinarum* cultures. A decrease in salinity from 28 PSU down to 15–20 PSU led to a significant increase in the BSi concentration in the cells of both microalgae species [68]. The differences in the BSi content in *Chaetoceros* spp. of the Siberian Arctic seas found in our study (Table 3) were probably associated with the response of the diatom complex of the phytoplankton community to abiotic environmental factors such as temperature, salinity, and/or local variability of mineral nutrition (Table 1), and, probably, some other factors [11,15,37].

Despite significant differences in the available data and the complexity of estimates of the true BSi content in diatoms from natural communities, it should be noted that this group of phytoplankton plays a key role in the biogeochemical cycle of silicon in the World Ocean [51,56,58]. The BSi production by diatom phytoplankton in the euphotic zone of the ocean ranges from 5.6 to 7.8 × 10^15^ g Si per year. On a global scale, ~50% of silicon is dissolved and recycled in the upper 100-m water layer, and ~2.8–3.9 × 10^15^ g of Si per year is exported to the deeper ocean as biogenic detritus [69].

### 4.2. Major Elements

Along with BSi, other essential macronutrients were alkaline (Na and K) and alkaline earth (Mg and Ca) metals, as well as biogenic (P and S) elements, with concentrations from hundredths to tenths of a percent of dry weight (Table 3). The greatest variability was noted for Na^+^ and K^+^ and the lowest for P and S, which was probably due to their involvement in fundamentally different metabolic processes in the cell. Alkali metals form the electrolyte environment of the body; compared to other elements, they are least capable of forming coordination bonds; however, they bind ligands, in which the oxygen atom acts as a donor (phosphates, carbon groups, and carbonyl groups). In an aqueous medium, Na^+^ and K^+^, as well as Rb^+^ and Cs^+^ from bound complexes, rapidly exchange and diffuse as simple ions [70]. On the contrary, phosphorus and sulphur are strongly associated with intracellular structures. They are part of the overwhelming number of bioorganic molecules and are involved in various metabolic processes; therefore, maintaining their relatively constant contents ensures the stability of cellular homeostasis [71,72]. In general, the variation in the concentration of major elements in the *Chaetoceros* spp. diatoms in the Siberian Arctic seas was probably associated with the different functional states of microalga communities in the autumn period and with the stages of their cell cycles [37,43]. However, this assumption requires additional studies. In the future, it may be supplemented after the comprehensive publication of the results of phytoplankton studies in the seas of the Siberian Arctic, which were carried out during cruise no. 69 of the R/V Akademik Mstislav Keldysh [39].

Overall, the average concentrations of all macronutrients in the *Chaetoceros* spp. diatoms were significantly lower compared to those in total phytoplankton and total plankton of the World Ocean (Table 4). In our opinion, some studies report “abnormally” high values (>5–10% DW) for alkali and alkaline earth metals [5,6,7]. This may be the result of contamination of samples with seawater salts and subsequent distortion of the true content of this group of elements in phytoplankton. In a previous study, it has been shown that it was the poor washing of total plankton samples from the White Sea that contributed to an increase in their ash content, mainly due to the presence of sodium [17].

### 4.3. Iron

Iron is an important essential trace element for microalgae. The obtained values and the range of their variation were in good agreement with previously published data for marine phytoplankton (Table 4).

Iron takes an active part in the processes of growth, development, photosynthesis, cellular respiration, assimilation of various forms of nitrogen, operating of the electron transport chain, and other biological processes involving energy transfer [8,75]. Fe deficiency prevents the complete biological use of nitrates from the environment and affects the species composition of phytoplankton. For various open-ocean areas, there is strong evidence that phytoplankton growth is limited by the abundance of biologically available forms of Fe [76]. At the same time, Fe supply in diatoms affects the efficiency of Si accumulation in cells. Diatoms lacking Fe more intensively extract silicic acid from the medium and form large numbers of silicified frustules. In various regions of the ocean, where diatoms predominate in the phytoplankton community and a deficiency of biologically available forms of Fe is observed, frustule silicification has global ecological and biogeochemical consequences [77]. On the one hand, more intense absorption of Si leads to the depletion of its content in surface waters and the formation of secondary Si-limiting of primary production. In this case, further diatom growth stops, even in the presence of other nutrients. On the other hand, silicified diatom cells have higher sink rates. This leads to an acceleration in the release of BSi and organic carbon to the bottom and a decrease in the efficiency of their recycling in the water column [78].

### 4.4. Aluminum

Currently, the biological role of aluminum is not fully understood. Traditionally, Al is considered to be a tracer of terrigenous material enriched by clay minerals [79,80] or indicating aeolian transport of dust or sand [81,82]. Its presence in plankton samples may indicate the presence of a trace admixture of clay particles [4,83,84]. For many living organisms, including marine phytoplankton, dissolved forms of aluminum may be toxic [85,86]. However, it is known that diatoms can extract Al from seawater and incorporate it into their cell walls [87]. At the same time, the role of this element in biomineralization has not been fully studied, nor has its effect on the structure of the formed BSi. Several studies reported that Al has been uptaken by the cell when forming the frustule [88]. Aluminum is included into the frustule during silicification, which suggests the structural incorporation of Al inside the silica framework synthesized by the organism. The incorporation of Al into the diatom frustule modifies its properties and affects the solubility of BSi [89,90]. Most notably, Al incorporation into the frustules of several diatom species leads to a significantly enhanced hydrolysis resistance and longer lifetime of frustules compared to Al-free ones [88,90,91].

In planktonic complexes of the open ocean, the primary uptake of Al as a result of BSi biosynthesis gives an Al: Si ratio as 10^−4^ to 10^−3^ [92]. The uptake of Al depends on the particular species and is limited to a certain value for each species [93]. For the *Chaetoceros* spp. diatoms, the Al:Si ratio varied from 1.7 × 10^−3^ (Kara Sea) to 8.9 × 10^−3^ (Laptev Sea), averaging 5.8 × 10^−3^. These ratios are at least one order of magnitude lower than those found in the dead diatoms isolated from the deep-sea bottom sediments [92]. Earlier, it was reported that secondary Al absorption took place after the diatom death, increasing the Al:Si ratio. Since the frustules of living diatoms are protected from the outer environment by biological membranes, secondary uptake of Al occurs most likely after their post-mortem destruction [94]. It is assumed that the formation of an aluminosilicate phase on the surface of the frustules is one of the mechanisms of the postmortem accumulation of Al in diatoms. Structural incorporation of Al into BSi takes place upon long contact of the cell with high concentrations of Al (as a rule, during sedimentation at the water/bottom interface) [90,94]. The Al:Si values obtained in the present study (5.8 × 10^−3^) may probably indicate the predominantly primary, intravital accumulation of Al by diatoms of the genus *Chaetoceros*. However, we do not exclude the possibility of the formation of various complexes on the surface of diatom cells, but probably their contribution was of minor importance. Both the lifetime and posthumous incorporation of Al into the frustules of diatoms reduce the solubility of BSi, thus affecting the efficiency of silica recycling in the ocean [6,11,20,77,91]. However, the intravital and postmortem accumulation of Al by diatoms is based on fundamentally different processes, which are biological uptake during biosynthesis by living diatoms versus inorganic uptake during the postmortem modification of the diatom frustules [94].

The biogeochemical cycles of Al and Si in the ocean are closely interrelated, since both elements enter the environment as a result of continental weathering [82,91]. This relationship is mainly determined by the large-scale biological cycles of diatoms, which provide up to 40% of the primary production in the ocean per year [69,95]. The assimilation of dissolved forms of Si and Al by diatoms and their subsequent sedimentation as biogenic silicon-containing detritus ensures the effective removal of elements from the upper photic layer of the ocean to its deeper parts and their further accumulation in bottom sediments [69,91,94]. Taking into account an increasing number of studies on the biological role of Al in diatoms and microalgae, it should be expected that our understanding of its biogeochemical cycle in the ocean may change [96].

### 4.5. Trace Elements

The data on the trace element composition of *Chaetoceros* spp. obtained in our study reflect a high variation in the accumulation of chemical elements, both individual and by the element group (Table 3). Since the chemical composition of *Chaetoceros* spp. from all three seas was studied, the differences obtained cannot be explained by the species-specific features of the accumulation of some trace elements, as observed earlier for zooplankton in the Laptev Sea [4] and the Kara Sea [84]. However, there are probably other factors or a combination of them responsible for this phenomenon. At first glance, high levels of Mn and Ti, as well as Al and Fe, in microalgae from the Laptev Sea (Table 3) may indicate that this sample contains a trace admixture of abiogenic seston, for example, clay particles [79,97]. However, no obvious source of such material can be named. The sampling was carried out at the stations located as far as possible from the areas strongly affected by the freshwater runoff bringing high content of suspended particulate matter (Figure 1) [35,39]. The ingress of clay particles as a result of the turbidity of bottom sediments may also be neglected, since no samples were obtained in the near-bottom layer. It should also be noted that a thorough sample preparation procedure was applied, which aimed at preventing contamination of samples with abiogenic detritus (Section 2.2, Field Studies). It should be noted that aeolian dust could also be a source of lithogenic tracers [82]. However, there are no data available at present in the literature on the aeolian transport of sedimentary matter in the seas of the Siberian Arctic and the bioavailability of metals associated with it Alongside with the latter, a number of studies report that various species of microalgae may accumulate high concentrations of Mn, Ti, Al, and Fe, both when grown in culture [3,98] and in the natural environment [5]. It is obvious that microalgae grown in culture are not contaminated with clays, and there is no reason why natural phytoplankton cannot also strongly concentrate these elements (the accumulation of Fe and Al is discussed above). It is important to note that *Chaetoceros* spp. from the Laptev Sea was characterized by a higher accumulation of all groups of elements (Table 3). We consider several factors that could have both individual and combined effects on the formation of the chemical composition of diatoms during the study period.

Firstly, the studied phytoplankton samples could be presented by natural populations of *Chaetoceros* spp., which were at different stages of the cell cycle. The differences in their chemical composition were probably associated with the unequal exchange rate of chemical elements between the cell and the environment, as well as with the peculiarities of the intravital and posthumous concentration of elements and their recycling. For example, similar patterns have been noted earlier for the zooplankton of the Kara Sea [84].

Secondly, the processes of adsorption and/or formation of hydroxyl complexes on the cell surface, which act together or separately, may be the mechanisms ensuring the uptake of elements by senescent phytoplankton [5]. It is known that some trace elements, including heavy metals (Cr, Mn, Fe, Ni, Cu, Zn, Cd, Pb, etc.), are easily adsorbed by certain types of organic material, for example, by the chitinous exoskeleton of planktonic crustaceans [84,99]. On the other hand, the biological uptake of elements and their further participation in biochemical processes inside the cell, as well as in the biosynthesis of the silicon frustule of diatoms, may be important mechanisms for the assimilation of elements in living, actively growing phytoplankton [8,10,14].

Thirdly, the uptake of trace elements by phytoplankton requires a chemical reaction between the other metal and transport ligands that are located on the cell surface [15]. As a result of biogeochemical evolution, various microorganisms and microalgae have developed various cellular mechanisms capable of rapidly and specifically assimilating trace elements from the environment [13,100]. The specificity of metals’ uptake is especially relevant for microorganisms and microalgae living in the metal-poor surface water layer of the ocean. Phytoplankton uptake experiments demonstrate competitive kinetics between biologically important divalent metals such as Zn^2+^, Cd^2+^, Co^2+^, Mn^2+^, and Fe^2+^. Deficiency of one metal may lead to increased absorption of others [101]. An increase in the uptake of Cd and Co by model cultures of diatoms *T. weissflogii* and *T. pseudonana* at low Zn concentrations in the medium may serve as an example of important consequence of this process [11,101]. In such experiments, when zinc content is limited in the medium but excessive Cd or Co is/are added, the biochemical basis for restoring high growth rates in diatoms is associated with the replacement of Zn^2+^ with Co^2+^ or Cd^2+^ in the active site of the carbonic anhydrase enzyme [102].

Therefore, in marine ecosystems, the abundance and bioavailability of essential elements in water and the patchiness of their distribution will have a significant impact on the formation of the multi-element composition of phytoplankton and its certain taxonomic groups [11,13].

### 4.6. Rare Earth Elements

Currently, there are no published data on the REE content in marine phytoplankton. There is only one publication on the concentrations of Sc, La, Ce, Sm, and Eu in the total phytoplankton of the coastal zone of Japan [7] (Table 4). Despite the importance of studying REEs in emerging issues of marine geochemistry, little is known about their accumulation in marine biota. The role of primary producers in the bioaccumulation and trophodynamics of REEs in marine ecosystems has not been fully investigated yet [22,103,104]. At the same time, the modern development of high technologies, and the creation of new composite materials and alloys precondition the intensive industrial use of a wide range of REEs [104,105]. Due to the potential ecotoxicological risks associated with their anthropogenic release into the environment, REEs have recently been identified as “new emerging pollutants” (EP) [103]. However, the EP content in marine biota is currently not regulated, since their toxicological effects in vitro and in situ are often poorly understood, and reliable quantitative analytical methods have not been available until recently [106].

Modern concepts of the biogeochemistry of REEs in marine ecosystems and the potential role of living organisms on their biogenic cycles do not allow us to talk unambiguously about the reasons for the variability of the accumulation of REEs in *Chaetoceros* spp. in the seas of the Siberian Arctic. However, it can be assumed that the contents of REE, as well as those of major and trace elements, are primarily preconditioned by the functional state of the phytoplankton community of the Siberian Arctic seas in the autumn period, by the cell developmental stage, and by the provision of mineral nutrition [11,15,39].

### 4.7. Global and Regional Levels of Chemical Element Contents in Phytoplankton: Issues of Estimating Background Concentrations

Table 4 shows the average multi-element composition of the *Chaetoceros* spp. diatoms from the natural complexes of the Siberian Arctic seas and its comparison with the literature data on the chemical composition of marine and oceanic phytoplankton as well as total plankton.

The extreme difficulties in estimating the average chemical composition of phytoplankton nowadays need to be considered for a number of reasons. First, obtaining pure samples of microalgae from natural phytoplankton communities is not an easy task, and in some cases, it is naturally impossible [2,5,7,17]. Second, significant spatial and temporal variability of the species composition of the phytoplankton community can make it difficult to compare the results of elemental analyses carried out for individual taxonomic groups and total samples [4,5,83]. Third, the presence of groups of microalgae with proven species-specific accumulation of macronutrients (for example, silicon in diatoms, calcium and strontium in Coccolithophoridae) have biogeochemical properties that affect the accumulation of other chemical elements [1,20,53,69]. Fourth, most works on the chemical composition of microalgae are focused on studying the accumulation of major and trace elements under artificial cultivation conditions, which significantly complicates the extrapolation of such data to natural complexes [14,51,90,96]. Fifth, despite the high interest in the study of the chemical composition of marine plankton, the content of a relatively small set of elements is usually assessed. This approach does not provide a complete understanding of the nature of the formation of the chemical composition of phytoplankton [3,7,19].

At present, the review by Savenko [16], published more than 30 years ago and based on the literature data of the 1960s to the 1980s, may be considered the most complete summary of the average elemental composition of various groups of oceanic plankton. In this article, the author critically summarizes the results of analytical determination of the contents of 63 chemical elements in ocean phytoplankton, zooplankton, and mixed (total) plankton, based on an analysis of 76 literature sources. Undoubtedly, these data are important and of great value. Considering the tremendous progress in the development of physicochemical analytics, today these data may be considered outdated, although no up-to-date alternative exists. Two other publications on the chemical composition of phytoplankton have been published even earlier [2,5], and therefore, there is urgent need for targeted studies on this issue, applying up-to-date methods and approaches. In this context, despite the long history of studying the elemental composition of phytoplankton and its individual ecological and systematic groups in situ and in vitro, it is obvious that the global knowledge is far from complete. There are no comprehensive reviews focusing on the formation of the chemical composition of phytoplankton and taking into account the species, spatial, temporal, and ontogenetic variability, as well as climatic zoning in the accumulation of a wide range of major and trace elements. At the same time, data on the contents of some REEs, belonging to new emerging pollutants, are completely absent for total phytoplankton and its particular groups [103,106].

## 5. Conclusions

Despite the long history of studying the chemical composition of marine phytoplankton, the first comprehensive understanding of the content of a wide range of major, trace, and rare-earth elements in the *Chaetoceros* spp. diatoms during the autumn season is provided by the present study. These data may be applied for the formation of databases of the chemical composition of marine living organisms, for assessing the dynamics and fluctuations of marine ecosystems in the context of climate changes in the Arctic Ocean, for environmental monitoring, for improving the cultivation methods of diatom microalgae, taking into account their multi-elements nutritional needs, and, finally, for the development of safety principles for using algae as potential natural sources of functional nutrition for humans, farm animals, and mariculture.

## Figures and Tables

**Table 1:** Coordinates, depth, salinity, and temperature of the sampling locations.

Station	Location	Date	Latitude °N	Longitude °E	Depth, m	Salinity, PSU		Temperature, °C	
						Surface	45-m	Surface	45-m
5587/2	KS	24.09.2017	74.7824	66.5917	189	25.22	33.99	3.46	−1.74
5625	LS	16.09.2017	76.7729	125.7942	70	22.83	34.02	2.55	−1.77
5612	ESS	08.09.2017	74.3833	168.1866	50	29.28	31.54	0.52	−1.66

Note: KS—Kara Sea, LS—Laptev Sea, ESS—East Siberian Sea.

**Table 2:** Detection limits and measured and certified values of element concentrations in Standard Reference Material.

Element	Detection Limit	Canadian Pondweed GSO 8921–2007 EK-1		Oriental Basma Tobacco Leaves INCT–OBTL–5		Polish Virginia Tobacco Leaves INCT–PVTL–6	
		Measured Value	Certified Value *	Measured Values	Certified Value *	Measured Values	Certified Value *
Major Elements (% DW)							
Na	0.0006	0.71	0.69 ± 0.05	0.025	0.044 **	0.008	0.006 **
Mg	0.0003	0.33	0.32 ± 0.02	0.85	0.853 ± 0.034	0.23	0.241 ± 0.009
P	0.0002	0.25	0.24 ± 0.03	0.17	0.170 ± 0.012	0.24	0.242 ± 0.015
S	0.0012	0.33	0.34 ± 0.05	0.44	0.455 ± 0.091	0.35	0.378 ± 0.059
K	0.0006	3.28	3.22 ± 0.16	2.28	2.271 ± 0.076	2.42	2.640 ± 0.090
Ca	0.0044	2.88	2.80 ± 0.17	3.94	3.996 ± 0.142	2.28	2.297 ± 0.078
Al	0.0012	0.099	0.099 ± 0.012	0.18	0.198 ± 0.028	0.029	0.025 ± 0.005
Fe	0.0012	0.25	0.26 ± 0.01	0.14	0.149 **	0.024	0.026 **
Trace Elements (µg g^–1^ DW)							
Li	0.04	1.50	1.44 ± 0.18	23.6	19.3 **	3.69	3.35 ± 0.67
Be	0.01	0.065	0.07 **	0.07	0.081 **	0.022	–
B	4	26.6	33 ± 10	34.4	33.6 ± 2.2	32.9	33.4 ± 1.9
Ti	2	49.4	77 ± 14	76.5	80.7 **	12.68	12.3 **
V	0.3	3.55	3.8 ± 0.4	4.0	4.12 ± 0.55	0.39	0.405 ± 0.06
Cr	0.4	4.73	5.1 ± 0.5	4.94	6.3 **	0.67	0.91 **
Mn	0.3	517	520 ± 30	179	180 ± 6	135	136 ± 5
Co	0.1	1.39	1.5 ± 0.1	0.93	0.98 ± 0.07	0.17	0.15 ± 0.01
Ni	0.5	3.58	3.7 ± 0.4	8.3	8.5 ± 0.49	1.47	1.49 ± 0.14
Cu	0.9	10.8	11.2 ± 0.4	9.74	10.1 ± 0.4	4.84	5.12 ± 0.2
Zn	0.3	19.1	20.6 ± 1.4	53.4	52.4 ± 1.8	44.49	43.6 ± 1.4
Ga	0.2	0.29	0.4 **	0.51	–	0.09	–
As	0.1	0.78	0.76 ± 0.02	0.78	0.67 ± 0.09	0.13	0.14 ± 0.01
Se	0.05	0.35	0.3 **	0.29	–	0.22	–
Rb	0.03	3.23	3.5 ± 0.3	22.9	19.1 ± 1	6.27	5.97 ± 0.28
Sr	0.3	170	174 ± 9	106.7	105 ± 5	135	133 ± 6
Mo	0.1	1.18	1.2 **	0.38	0.41 ± 0.06	0.42	0.4 ± 0.03
Ag	0.02	0.012	0.017	0.048	0.053 ± 0.011	0.018	0.019 ± 0.004
Cd	0.01	0.083	0.1 ± 0.02	2.67	2.64 ± 0.14	2.18	2.23 ± 0.12
Sn	0.1	0.15	0.12 **	0.13	–	0.05	0.031 **
Sb	0.02	0.072	0.08 ± 0.02	0.054	0.076 ± 0.013	0.035	0.037 ± 0.004
Cs	0.005	0.1	0.108 ± 0.008	0.29	0.288 ± 0.02	0.025	0.026 **
Ba	0.3	77.9	78 ± 7	62.6	67.4 ± 3.8	42.9	41.6 ± 1.9
Hg	0.04	0.017	0.03 **	0.018	0.021 ± 0.001	0.022	0.023 ± 0.002
Tl	0.001	0.016	0.02 **	0.052	0.051 **	0.025	0.023 **
Pb	0.2	1.12	1.1 ± 0.1	1.93	2.0 ± 0.3	0.82	0.97 ± 0.15
Bi	0.003	0.018	0.023 **	0.09	–	0.145	0.14 **
Th	0.02	0.38	0.4 **	0.48	0.5 ± 0.04	0.085	0.089 ± 0.007
U	0.004	1.42	1.4 ± 0.1	0.095	0.113 **	0.02	0.022 **
Rare-Earth Elements (µg g^–1^ DW)							
Sc	0.01	0.35	0.38 ± 0.02	0.6	0.64 ± 0.027	0.19	0.06 ± 0.003
Y	0.01	1.18	1.3 **	0.98	0.963 **	0.22	0.218 **
La	0.003	2.03	2.1 ± 0.1	1.58	1.7 ± 0.1	0.50	0.54 ± 0.027
Ce	0.008	3.58	3.4 ± 0.3	2.84	3.0 ± 0.2	0.69	0.743 ± 0.051
Pr	0.001	0.41	0.42 **	0.33	0.321 **	0.08	0.083 **
Nd	0.006	1.56	1.6 ± 0.2	1.28	1.3 ± 0.1	0.32	0.32 ± 0.02
Sm	0.001	0.29	0.31 ± 0.03	0.25	0.26 ± 0.01	0.055	0.058 ± 0.004
Eu	0.006	0.04	0.047 ± 0.008	0.05	0.06 ± 0.004	0.011	0.014 ± 0.003
Gd	0.007	0.265	0.35 ± 0.08	0.23	0.243 **	0.052	–
Tb	0.005	0.038	0.041 ± 0.005	0.034	0.035 ± 0.002	0.007	0.008 ± 0.001
Dy	0.006	0.21	0.36 ± 0.13	0.185	0.184 **	0.037	–
Ho	0.001	0.04	0.47 ± 0.008	0.035	0.035 **	0.007	–
Er	0.001	0.12	0.13 ± 0.02	0.1	0.1 ± 0.01	0.0187	0.019 ± 0.003
Tm	0.002	0.015	0.021 ± 0.007	0.013	0.014 **	0.0024	–
Yb	0.007	0.1	0.074 ± 0.006	0.087	0.115 ± 0.02	0.015	0.028
Lu	0.003	0.015	0.019 ± 0.003	0.012	0.017 **	0.0024	–

Note: dash—no data; *—mean ± standard deviation; **—information values.

**Table 3:** Concentrations of chemical elements in the diatom *Chaetoceros* spp. from the Siberian seas, the Russian Arctic.

Element	The Kara Sea	The Laptev Sea	The East-Siberian Sea	Mean ± SE
Major Elements (% DW)				
Na	0.09	0.28	0.78	0.38 ± 0.21
Mg	0.11	0.19	0.17	0.16 ± 0.03
P	0.27	0.39	0.50	0.39 ± 0.07
S	0.37	0.53	0.50	0.46 ± 0.05
K	0.02	0.12	0.32	0.15 ± 0.09
Ca	0.30	0.20	0.18	0.22 ± 0.04
Si	18.11	20.12	19.05	19.10 ± 0.58
Al	0.03	0.18	0.13	0.11 ± 0.04
Fe	0.07	0.35	0.14	0.19 ± 0.08
Sum	19.37	22.36	21.77	21.17 ± 1.0
Trace Elements (μg g^−1^ DW)				
Li	3.46	1.37	1.53	2.12 ± 0.67
Be	0.01	0.07	0.04	0.04 ± 0.01
B	45.6	37.0	37.6	40.1 ± 2.76
Ti	104	123	112	113 ± 5.42
V	3.5	6.2	2.9	4.19 ± 1.04
Cr	3.4	8.7	3.6	5.24 ± 1.73
Mn	17.5	108	129	84.7 ± 34.1
Co	0.24	1.08	0.84	0.72 ± 0.25
Ni	2.0	6.2	3.6	3.95 ± 1.22
Cu	14.6	18.0	12.0	14.88 ± 1.76
Zn	139	342	273	251.67 ± 59.48
Ga	<d/l	<d/l	<d/l	-
As	2.2	3.3	3.3	2.89 ± 0.33
Se	0.75	<d/l	2.0	1.38
Rb	0.50	3.3	3.3	2.38 ± 0.94
Sr	24.9	66.6	25.5	39.0 ± 13.8
Mo	1.7	1.2	0.51	1.15 ± 0.35
Ag	0.044	0.041	0.046	0.044 ± 0.002
Cd	0.3	0.57	1.31	0.73 ± 0.3
Sn	1.1	1.6	1.5	1.41 ± 0.14
Sb	0.12	0.49	0.18	0.26 ± 0.11
Cs	0.026	0.17	0.14	0.11 ± 0.04
Ba	45.9	84.6	95.5	75.33 ± 15.04
Hg	0.11	0.09	0.09	0.09 ± 0.006
Tl	0.21	0.02	0.012	0.082 ± 0.07
Pb	1.8	7.8	3.6	4.4 ± 1.77
Bi	0.025	0.043	0.019	0.03 ± 0.007
Th	0.051	0.28	0.15	0.16 ± 0.07
U	10.02	4.11	1.08	5.08 ± 2.68
Sum	423.07	825.83	714.35	654.42 ± 120.07
Rare Earth Elements (μg g^−1^ DW)				
Sc	0.45	0.92	0.91	0.76 ± 0.16
Y	0.12	0.56	0.35	0.34 ± 0.13
La	0.22	0.98	0.68	0.63 ± 0.22
Ce	0.52	2.1	1.2	1.25 ± 0.44
Pr	0.05	0.21	0.12	0.13 ± 0.05
Nd	0.25	0.97	0.60	0.61 ± 0.21
Sm	0.034	0.17	0.096	0.1 ± 0.04
Eu	0.009	0.11	0.025	0.047 ± 0.03
Gd	0.034	0.16	0.095	0.095 ± 0.04
Tb	<d/l	0.012	<d/l	-
Dy	0.028	0.12	0.073	0.074 ± 0.03
Ho	0.005	0.023	0.014	0.014 ± 0.005
Er	<d/l	0.056	0.021	0.038
Tm	<d/l	0.009	<d/l	-
Yb	0.010	0.062	0.036	0.036 ± 0.02
Lu	<d/l	0.007	<d/l	-
Sum	1.73	6.47	4.22	4.14 ± 1.37

Note: <d/l—below detection limit.

**Table 4:** A comparison of the multi-element composition of the diatom *Chaetoceros* spp. (the Russian Arctic seas) and marine/ocean phytoplankton and marine/ocean total plankton.

Element	*Chaetoceros* spp. ^a^	Total Phytoplankton			Total Plankton				
		Coastal Areas ^b^	Marine ^c^	Ocean ^e^	The White Sea ^f^	The Baltic Sea ^g^	The Sea of Japan ^h^	Ocean	
								^e^	^i^
in % DW									
Na	0.38	0.05–5.72	8.85–13.83	3.0	5.3	3.9	3	3.5	3.3
Mg	0.16	-	1.1–1.64	0.8	-	0.67	-	1.1	9.4
P	0.39	-	-	1.0	-	0.25	-	0.8	0.28
S	0.46	-	-	0.5	-	-	-	0.55	0.83
K	0.15	-	1.1–1.33	1.2	1.2	1.01	-	0.9	5.2
Ca	0.22	-	0.53–0.65	0.45	1.1	1.21	1.5	1.9	1.4
Si	19.10	-	4.68–7.01	8.0	-	-	-	6.0	0.15
Al	0.11	0.23–1.75	0.004–0.04	0.01	-	0.27	-	0.01	0.0062
Fe	0.19	0.06–0.75	0.02–0.15	0.09	0.16	0.36	0.3	0.08	0.016
in µg g^−1^ DW									
Li	2.12	-	-	50	-	5.9	-	40	5
Be	0.04	-	-	0.6	-	-	-	0.4	0.003
B	40.1	-	-	30	-	-	-	50	120
Sc	0.76	0.4–2.43	-	-	0.28	0.76	0.19	0.2	0.07
Ti	113	-	27	100	-	350	-	50	11.0
V	4.19	13.4–38.5	3–5 ^d^	4	3.8	8.5	-	4	3.5
Cr	5.24	17.2–51.9	3.9	10	218.3	27.3	54.7	10	1.8
Mn	84.7	17–216	6.1–13.3	10	62.5	600	-	10	20
Co	0.72	0.24–1.83	38 ^d^	1.5	0.86	18	0.23	1.5	0.43
Ni	3.95	-	1.9–7.8	10	4.1	35	16.2	10	1.4
Cu	14.88	-	3.2–14.8	60	75.3	21	7	40	12
Zn	251.7	12–362	19–122	300	360	140	8.7	300	39
Ga	< d/l	-	-	0.2	1	-	-	0.2	0.5
As	2.89	3.3–9.6	12–36 ^d^	14	12.3	4.2	1.16	10	15
Se	1.38	1.3–4.32	3.5 ^d^	4	0.45	1.8	-	4	0.06
Rb	2.37	-	-	3	5.75	6	-	3	1.8
Sr	39.0	75–13,100	119–697	390	110	190	-	300	1100
Y	0.34	-	-	-	0.1	-	-	4	-
Mo	1.15	-	-	0.7	0.18	10	13.1	1	0.39
Ag	0.044	-	0.2–0.6	0.2	-	2.7	-	0.4	0.22
Cd	0.73	-	1.5–3.9	3	2.4	2	6.7	3	0.72
Sn	1.41	-	-	10	-	-	-	8	0.29
Sb	0.26	0.95–2.44	-	0.1	1.5	0.5	-	0.1	0.16
Cs	0.11	0.18–1.26	0.11 ^d^	0.03	0.25	0.32	1.06	0.04	0.072
Ba	75.33	666–1756	19–287	80	22	800	-	100	19
La	0.63	1.05–8.27	-	-	0.73	4.2	5.6	0.8	0.14
Ce	1.25	1.07–8.27	-	-	1.46	10	10.3	1.2	0.23
Pr	0.13	-	-	-	-	-	-	0.15	-
Nd	0.61	-	-	-	-	4.4	-	0.7	-
Sm	0.1	0.15–0.88	-	-	0.1	0.61	0.53	0.07	-
Eu	0.047	0.034–0.207	-	-	0.021	0.14	0.15	0.02	-
Gd	0.095	-	-	-	-	-	-	0.2	-
Tb	0.012	-	-	-	0.014	0.13	0.9	0.3	-
Dy	0.074	-	-	-	-	-	-	0.15	-
Ho	0.014	-	-	-	-	-	-	0.03	-
Er	0.038	-	-	-	-	-	-	0.09	-
Tm	0.009	-	-	-	-	-	-	0.015	-
Yb	0.036	-	-	-	0.058	0.29	-	0.07	-
Lu	0.007	-	-	-	0.008	0.04	-	0.015	-
Hg	0.09	-	0.16–0.19	0.1	0.034	0.24	-	0.2	0.03
Tl	0.082	-	-	-	-	-	-	-	-
Pb	4.4	-	7.2–9.2	20	16.6	25	10.2	20	8.7
Bi	0.03	-	-	-	-	-	-	-	-
Th	0.16	0.25–1.06	0.42 ^d^	-	0.21	0.87	0.44	0.1	0.1
U	5.08	1.4–9.4	0.7 ^d^	0.7	-	0.41	-	0.6	0.8

Note: dash—no data; ^a^—our data for diatom *Chaetoceros* spp.; ^b^—[7]; ^c^—[5]; ^d^—[2]; ^e^—[16]; ^f^—[73]; ^g^—[74]; ^h^—[18]; ^i^—[6].

## Data Availability

The dataset is presented in this study.
